# Diagnostic dilemma in female genital tuberculosis- staining techniques revisited

**Published:** 2013-07

**Authors:** Bineeta Kashyap, Namita Srivastava, Iqbal R Kaur, Rajat Jhamb, Deepak K Singh

**Affiliations:** 1 *Department of Microbiology, University College of Medical Sciences, Guru Teg Bahadur Hospital, New Delhi, India.*; 2 *Department of Medicine, University College of Medical Sciences, Guru Teg Bahadur Hospital, New Delhi, India.*; 3 *Department of Pathology, University College of Medical Sciences, Guru Teg Bahadur Hospital, New Delhi, India.*

**Keywords:** *Culture*, *Tuberculosis*, *Genital*, *Female*

## Abstract

**Background:** Tuberculosis (TB) is an increasing public health concern worldwide. On a global scale it has a devastating impact in developing nations. Genital TB, an extrapulmonary form, is not uncommon particularly in areas where pulmonary TB is prevalent. Genital TB may be asymptomatic or may even masquerade as other gynaecological conditions; hence, diagnosis requires a high degree of suspicion and the use of appropriate investigations.

**Objective:** This study attempted to identify endometrial TB in endometrial biopsies taken from women evaluated for infertility by comparison of various staining techniques.

**Materials and Methods:** A comparative cross sectional study was conducted from February 2011 to April 2011 in Guru Teg Bahadur Hospital, New Delhi. Endometrial biopsy specimens from 55 endometrial TB suspects were stained for acid fast bacilli by Ziehl Neelson staining and Gabbet staining. The biopsy samples were also subjected to Auramine Phenol fluroscent staining and H and E staining. Culture on Lowenstein Jensen medium was taken as the gold standard.

**Results:** Three samples were culture positive giving positivity rate of 5.4%. Considering culture as the gold standard the senstivities of ZN, Gabbet, fluorescent and H and E staining were 33, 33, 66, and 66% respectively while their specificities were 100, 100, 98, and100% respectively.

**Conclusion:** Combination of fluorescent staining techniques along with one of the acid fast staining techniques or histopathology achieves sufficient sensitivity and specificity for the diagnosis of female genital tuberculosis. There is an urgent need for developing definitive diagnostic methods to make a conclusive diagnosis of genital TB.

## Introduction

Tuberculosis (TB), once thought to be a disease of poor countries, has resurged worldwide and become a major public health concern, with about six million new cases a year. *Mycobacterium tuberculosis* has infected nearly one-third of the world’s population leading the WHO to declare tuberculosis a global emergency in 1994. On a global scale it has a devastating impact in developing nations (1). A report of the WHO shows that there are at present, 20 million TB patients in the world, of whom approximately three-quarters, that is 15 million, live in developing countries (2, 3).

TB occurs in two forms; pulmonary and extra pulmonary TB. Female genital TB is an uncommon form of extra pulmonary TB of which infertility is the commonest symptom (4, 5). Tuberculosis of female genital tract; though uncommon in western world, is still prevalent in developing countries like India (6). Genital TB in females is found in 0.75-1% of gynecological admissions in India with considerable variation from place to place. The disease is responsible for 5% of all female pelvic infections and occurs in 10% and 15-20% of cases of pulmonary and extra pulmonary tuberculosis respectively (7).

Female genital tuberculosis is almost always secondary to a tubercular lesion elsewhere in the body. In descending order of frequency primary focus is often situated in lungs, lymph nodes, urinary tract, and bone and joints. The primary focus is most often healed or quiescent by the time the genital TB becomes active (8). 

Probability of a history of extra genital TB lesion in the form of calcified abdominal glands, mycobacterium tuberculosis in the urine or X-ray appearance of pleurisy or past or present pulmonary TB stays in cases of genital TB and a study has reported as high as 13% of pulmonary TB cases that had genital tuberculosis (9).

Genital TB usually remains silent and physical signs are usually not present definitively, hence the disease remains largely undiagnosed or specific investigations are not undertaken to rule out the problem. In premenopausal women, infertility is the leading concern. The reported incidence of infertility among women with genital TB is 58-87% (10). When at all present, symptoms are usually mild and local which include pelvic pain, menstrual disorders, vaginal bleeding and/or poor general health.

Endometrial TB may be asymptomatic or may even masquerade as other gynecological conditions. Its discovery is usually made unexpectedly, most often via an endometrial biopsy in the course of investigative studies into infertility. Diagnosis is achieved most effectively through a combination of high index of suspicion, thorough initial clinical assessment and the use of appropriate investigations. Female genital TB, being non-infectious, has been neglected by healthcare providers, but is an important cause of both significant morbidity and short- and long-term sequelae for the affected women. 

Histology demonstrates the typical caseous granulomatous lesion, highly suggestive of TB though not diagnostic; as a variety of infectious and noninfectious conditions (fungal infections and sarcoidosis etc.) can be associated with granuloma formation (11). Microscopy for acid fast bacilli can provide a quicker diagnosis though with variable sensitivities. Culture can give positive results for *Mycobacterium tuberculosis* in 4 weeks but can take up to 12 weeks. Besides technical drawbacks in demonstrating *M. tuberculosis *in the laboratory, a substantial number of TB lesions of the genital tract are bacteriologically mute; the infection being pauci-bacillary form of the disease. 

The aim of this study was to see the correlation of various staining techniques for acid fast bacilli and histopathology when compared to culture of Mycobacterium tuberculosis for the diagnosis of female genital tuberculosis.

## Materials and methods

A comparative cross-sectional study was conducted from February 2011 to April 2011 in the Mycobacteriology Laboratory, Department of Microbiology, University College of Medical Sciences, Guru Teg Bahadur Hospital, a tertiary care hospital in East Delhi Hospital. 55 cases clinically diagnosed with unexplained primary infertility were enrolled in the study after obtaining informed consent. The study adhered to the Helsinki Declaration. No conflict of interest was there.

Endometrial biopsy samples were collected by curetting the endometrial cavity by a small curette with or without dilating the cervix under deep sedation or anaesthesia, in the pre-menstrual phase of the cycle. Endometrial specimens were aliqueted into two portions-one kept in normal saline for microscopy and culture, and the other in formal saline for histopathology. Endometrial biopsy specimens were screened for acid fast bacilli by ZN staining and Gabbet staining. Fluorescent staining was done for all the samples. The samples were further subjected to histopathological examination and culture on Lowenstein Jensen (LJ) medium. Culture was taken as the gold standard. The criteria validity of the staining techniques and histopathology was evaluated with culture as the gold standard.

**Exclusion criteria**

Exclusion criteria were as follows: females with proven diagnosis of other causes of infertility, patients, who had granulomas on biopsy but later on proved to be of non-tuberculosis origin, and patients with previous history of anti-tubercular drug (ATD) intake.

**Staining for acid fast bacilli**

**Figure 1:**
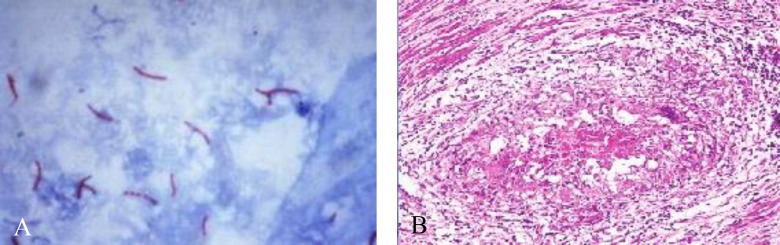
Acid fast bacilli seen in endometrial biopsy specimen on ZN staining (A), Caseating granulomas s/o TB (B) after H & E staining.

Ziehl-Neelsen (Z-N) staining was done by the conventional method (Figure 1). Gabbett's staining (GS): Endometrial biopsy smears were air-dried and heat-fixed (12). The slides were then flooded with 1% carbol-fuchsin stain (10 g of basic fuchsin, 100 mL of ethanol 95%, and 50 g of phenol).

This was allowed to stand at room temperature for 10 min. The smears were then washed in running water. They were then counterstained with Gabbett's methylene blue (1 g of methylene blue, 20 mL of sulfuric acid, 30 mL of 95% ethanol, and 50 mL of distilled water) for 2 min. The slides were then washed and air-dried.

**Culture**

Homogenized samples were cultured on Lowenstein Jensen egg medium for acid fast bacilli. They were incubated for 3-8 weeks. These culture slants were inspected for growth every week. Cultures were identified on the basis of colony morphology and biochemical tests. 

**Histopathology**

Paraffin embedded tissue sections were prepared from the biopsy specimens. The sections were stained with hematoxylin-eosin stain. The smears were examined by a pathologist for presence of granulomas suggestive of mycobacterium disease. For the diagnosis of genital tract TB, caseating granuloma was required to be present in the pathological specimens which included the epithelioid cells, giant cells, fibrosis, and proliferation of lymphocytes associated with caseous necrosis (Figure 1). 

**Fluorescent staining method (FS)**

Endometrial biopsy smears were air-dried and heat-fixed. The slides were flooded with freshly filtered auramine-phenol (add 100mL of conc auramine phenol to 900 ml of distilled water) for 10 min without heating. The smears were then washed in running water. The smears were then decolourized with a 1% acid/alcohol solution for 5 min. The slides were washed in running water and counterstained with a 0.1% potassium permanganate solution for 15 sec. This was followed by washing in running water and air-drying. 

**Statistical analysis**

This was a comparative cross sectional study, the criteria validity of the staining techniques and histopathology were found out with culture as the gold standard.

## Results

Three samples were culture positive giving positivity rate of 5.4%. Of the three culture positives, ZN and gabbett staining were positive in 1 case each while fluorescent staining and histopathology were positive in two out of three culture positive cases. Considering culture as the gold standard the senstivities of ZN, Gabbet, fluorescent and H and E staining were 33, 33, 66, and 66% respectively while their specificities were 100, 100, 98, and 100% respectively (Table I).

**Table I:** Correlation of Bacteriological and Histopathological diagnosis in female genital tuberclusis

**Staining used**	**sensitivity**	**specificity**	**Positive predective value**	**Negative predective value**
Ziehl Neelsen staining	33%	100%	100%	93%
Gabbett staining	33%	100%	100%	93%
Fluorescent staining	66%	98%	66%	98.11%
Haemotoxylin and Eosin staining	66%	100%	100%	98.14%

## Discussion

Endometrial TB is a known cause of infertility in women which, because of the global increase in the spread of TB, should always be considered when investigating the cause of infertility. Actual frequency of female genital tuberculosis is unknown despite different published data from various countries as it is often discovered incidentally or remains ‘undetected’ in symptomless patients. Genital tuberculosis is usually an indolent infection and takes years to manifest clinically after initial seeding. 

In India variable incidences of genital tuberculosis as a cause of infertility have been reported over the years (13). Although haematological studies, roentgenographic examination, hysterosalpingography, cytology, ultrasonongraphy and laproscopy can be useful aids in the detection of genital TB; the eventual diagnosis depends upon the characteristic histopathological picture confirmed by bacterial culture. However, as the primary lesion is often healed and inconspicuous by the time the disease is diagnosed, routine screening tests for pulmonary TB like chest roentgenographic findings, tuberculin test and sputum examination are not always abnormal (14). 

Gupta *et al* studied 40 infertile women with genital tuberculosis and found pulmonary tuberculosis in 9 of them and positive Mantoux test results in two patients (6). Histological findings in endometrial TB, in addition to being non-specific, depend on the cycle stage when the biopsy was taken and also on the site in the uterus in which it occurs (15). The specimens should be taken from the cornua in the premenstrual phase, allowing for the development of the typical granulomata. Tubercles may be missed unless multiple levels of curettage are examined (16). 

In our case also out of the 3 culture positive cases, only 2 showed caseating granulomas. As reported earlier also, caseation is rare in women of reproductive age group in tuberculous endometritis because of the fact that due to periodical loss of enometrium of menstruation the granulomas did not remain within the endometrial tissue long enough for caseation to develop and the disease process had to regenerate after menstrual shedding from the basal layer with start of each menstrual cycle (3). 

The gold standard remains the demonstration of acid-fast bacilli in biological specimens or culture. Microscopic examination of acid-fast bacilli requires presence of at least 10^4^ organisms/ml in the sample, while culture requires as little as 10^2^ organisms/ml. In addition to the non-availability of culture facilities in many laboratories, *M. tuberculosis* may take long incubation time (up to 8 weeks) to grow in LJ medium. Besides technical drawbacks in demonstrating *M. tuberculosis* in the laboratory, a substantial number of TB lesions of the genital tract are bacteriologically mute.

Smear microscopy for acid fast microscopy, though, is a low cost procedure suited to developing countries; the Z-N staining method is cumbersome and less sensitive as already mentioned previously. To achieve success in tuberculosis control in developing countries, modifications to eliminate technical and administrative problems in an affordable and practical way should be considered. A desire to develop an alternate staining procedure has resulted in several modifications of the traditional Z-N staining method; one of which is the cold staining using Gabbet’s methylene blue as a decolorizer and counter stain. The Gabbet cold staining method used in our study faired equally well when compared to the ZN method. 

The ease of performance, cost effectiveness and time saved give the cold staining method an added advantage. It is obvious from our study that a good correlation between histopathological and bacteriological examinations is essential for the diagnosis of all cases of genital tuberculosis. There is an urgent need for developing definitive diagnostic methods and criteria to be applied to make a conclusive diagnosis of genital TB and implemention of only a single of any of these could miss a substantial number of cases. In a recent study done in south India on genital TB, AFB staining gave the lowest detection rate of 5.2% followed by culture that also gave a low detection rate of 7.8%. 

The authors also did not find histopathology (with a positivity of only 11.05%) alone satisfactory as out of the seven samples that were positive by histology, four were not supported by PCR and classical granuloma formation found only in one (17). Genital tuberculosis is an elusive diagnosis and a high index of suspicion is the first step in the diagnostic process. Nonspecific clinical presentation, inefficacy of laboratory diagnostic tests, and inaccessibility of reproductive clinics have resulted in underreporting of Female Genital TB. In communities where tuberculosis is still a major health problem, it is imperative to consider the possibility of TB in women in the reproductive age group who present with the symptoms of infertility, chronic pelvic pain and menstrual dysfunction, where other causes have been excluded. 

Failure to do so may result in unnecessary and ineffective interventions in addition to various complications like increase of drug resistant TB, low conception rates, increase incidences of ectopic pregnancy and miscarriage rates. Therefore, a high index of suspicion for genital tuberculosis is essential while investigating infertile patients in communities where TB is still a prevalent disease.

## Conclusion

Combination of fluorescent staining techniques along with one of the acid fast staining techniques or histopathology achieves sufficient sensitivity and specificity for the diagnosis of female genital tuberculosis. There is an urgent need for developing definitive diagnostic methods to make a conclusive diagnosis of genital TB. 

## Conflict of interest

None Declared.
